# Sources of variability in quantification of CMR infarct size and their impact on sample size calculations - reproducibility among three core laboratories

**DOI:** 10.1186/1532-429X-17-S1-P84

**Published:** 2015-02-03

**Authors:** Igor Klem, Einar Heiberg, Lowie van Assche, Galen Wagner, Michele Parker, Han W Kim, John D Grizzard, Håkan Arheden, Raymond Kim

**Affiliations:** 1 Department of Clinical Physiology, Lund University, Lund University Hospital, Lund, Sweden; 2 Radiology, Virginia Commonwealth University Health Systems, Richmond, VA, USA; 3 Cardiology/Cardiovascular MRI Center, Duke University Medical Center, Durham, NC, USA; 4 Radiology, Duke University Medical Center, Durham, NC, USA

## Background

Infarct size is increasingly used as an efficacy endpoint in randomized trials comparing acute myocardial infarct (AMI) therapies. Infarct size, depicted by delayed-enhancement-CMR, is quantified using manual planimetry (MANUAL), visual scoring (VISUAL), or automated techniques using signal-intensity thresholding to define infarct borders (AUTO). Although AUTO is considered the most reproducible, prior studies did not account for the subjective determination of endocardial/epicardial borders, which all methods require. For MANUAL and VISUAL, prior studies have not explicitly defined how to treat intermediate signal-intensities due to partial volume. We wanted to assess sources of variability among 6 methods in quantification of AMI size, and illustrate the significance of these findings on sample size calculations for clinical trials.

## Methods

Scans of 30 AMI patients and 12 controls were sent to 3 core-laboratories. Infarct size was measured using 6 methods, each separated by >2-months time, as follows (n=540 evaluations): [1] AUTO; [2] AUTO-UC (user correction for endocardial border pixels, no-reflow, etc.); [3] MANUAL; [4] MANUAL-ISI (adjustment for intermediate signal-intensities); [5] VISUAL; [6] VISUAL-ISI. Reproducibility was assessed by calculating the coefficient of variation (CV) and intraclass correlation coefficient (ICC). Using standard variance components analysis, we calculated the variance between-patients and within-patients separately.

## Results

Mean infarct size varied between 16.8% and 27.2% of LV mass depending on the method. Even AUTO (no user interaction for infarct borders) resulted in significant within-patient variability given the need to delineate endocardial/epicardial contours (CV=10.6%). Adding user input to correct computer generated infarct borders resulted in a mild improvement in reproducibility (AUTO-UC: CV=8.3%; p=0.045 for comparison with AUTO). For manual and visual categories, explicitly adjusting for intermediate signal-intensities led to improved reproducibility (MANUAL-ISI vs MANUAL: CV=8.3% vs 14.4%; p=0.03; VISUAL-ISI vs VISUAL: CV=8.4% vs 10.9%; p=0.01). When the best techniques in each category were compared, reproducibility was similar (AUTO-UC, MANUAL-ISI, and VISUAL-ISI: CV=8.3%, 8.3%, 8.4%, respectively). For these 3 techniques the within-patient variability due to the quantification method was less than 10% of the total variability. Hence, there were minimal differences between these methods in the calculated sample sizes needed to detect a 3%, 5%, and 7% absolute reduction in acute infarct size.

## Conclusions

Among CMR core-laboratories, an important source of variability in infarct size quantification is the subjective delineation of endocardial/epicardial borders. When intermediate signal intensities are considered in manual planimetry and visual scoring, reproducibility and impact on sample size are similar to automated techniques.

## Funding

N/A.

**Table 1:** Summary of Reproducibility Analysis

	CV	ICC
AUTO	10.6%	0.91 [0.86, 0.95]

AUTO-UC	8.3%	0.96 [0.93, 0.98]

MANUAL	14.4%	0.87 [0.79, 0.93]

MANUAL-ISI	8.3%	0.94 [0.90, 0.97]

VISUAL	10.9%	0.85 [0.77, 0.92]

VISUAL-ISI	8.4%	0.90 [0.84, 0.95]

CV=coefficient of variation

ICC=intraclass correlation coefficient, 95% CI in parenthesis

